# Adverse Impact of *Desulfovibrio* spp. and Beneficial Role of *Anaerostipes* spp. on Renal Function: Insights from a Mendelian Randomization Analysis

**DOI:** 10.3390/nu12082216

**Published:** 2020-07-25

**Authors:** Mohsen Mazidi, Niloofar Shekoohi, Adrian Covic, Dimitri P. Mikhailidis, Maciej Banach

**Affiliations:** 1 Department of Twin Research and Genetic Epidemiology, King’s College London, St Thomas’ Hospital, Strand, London SE1 7EH, UK; 2 Department of Cellular and Molecular Nutrition, School of Nutritional Sciences and Dietetics, Tehran University of Medical Sciences, Tehran 14155-6446, Iran; shekoohi@gmail.com; 3 Nephrology Clinic, Dialysis and Renal Transplant Center, ‘C.I. PARHON’ University Hospital, and ‘Grigore T. Popa’ University of Medicine, 700469 Iasi, Romania; accovic@gmail.com; 4 Department of Clinical Biochemistry, Royal Free Campus, University College London Medical School, University College London (UCL), London NW3 2QG, UK; mikhailidis@aol.com; 5 Department of Hypertension, Chair of Nephrology and Hypertension, Medical University of Lodz, 93-338 Lodz, Poland; maciejbanach77@gmail.com; 6 Polish Mother’s Memorial Hospital Research Institute (PMMHRI), 93-338 Lodz, Poland; 7 Cardiovascular Research Centre, University of Zielona Gora, 65-046 Zielona Gora, Poland

**Keywords:** mendelian randomization, microbiota, chronic kidney disease

## Abstract

Background: The microbiota composition is now considered as one of the main modifiable risk factors for health. No controlled study has been performed on the association between microbiota composition and renal function. We applied Mendelian randomization (MR) to estimate the casual impact of eight microbiota genera on renal function and the risk of chronic kidney disease (CKD). Methods: MR was implemented by using summary-level data from the largest-ever genome-wide association studies (GWAS) conducted on microbiota genera, CKD and renal function parameters. The inverse-variance weighted method (IVW), weighted median (WM)-based method, MR-Egger, MR-Robust Adjusted Profile Score (RAPS), MR-Pleiotropy RESidual Sum and Outlier (PRESSO) were applied. A sensitivity analysis was conducted using the leave-one-out method. Results: The *Anaerostipes* genus was associated with higher estimated glomerular filtration rate (eGFR) in the overall population (IVW: β = 0.003, *p* = 0.021) and non-diabetes mellitus (DM) subgroup (IVW: β = 0.003, *p* = 0.033), while it had a non-significant association with the risk of CKD and eGFR in DM patients. Subjects with higher abundance of *Desulfovibrio* spp. had a significantly lower level of eGFR (IVW: β = −0.001, *p* = 0.035); the same results were observed in non-DM (IVW: β = −0.001, *p* = 0.007) subjects. *Acidaminococcus*, *Bacteroides*, *Bifidobacterium*, *Faecalibacterium*, *Lactobacillus* and *Megamonas* had no significant association with eGFR in the overall population, DM and non-DM subgroups (IVW: *p* > 0.105 for all groups); they also presented no significant association with the risk of CKD (IVW: *p* > 0.201 for all groups). Analyses of MR-PRESSO did not highlight any outlier. The pleiotropy test, with very negligible intercept and insignificant *p*-value, also indicated no chance of pleiotropy for all estimations. The leave-one-out method demonstrated that the observed links were not driven by single single-nucleotide polymorphism. Conclusions: Our results suggest an adverse association of *Desulfovibrio* spp. and a beneficial association of *Anaerostipes* spp. with eGFR. Further studies using multiple robust instruments are needed to confirm these results.

## 1. Introduction

In humans, up to 100 trillion bacterial cells from approximately 500 distinct species are present, and the gastrointestinal (GI) tract is the main habitat for >70% of those microbes [1,2]. Importantly, the microbiome can influence human health and well-being, with the potential to affect human physiology both in health and in disease [1]. A healthy gut microbiota exerts positive effects by contributing to energy metabolism [3] and micronutrient homeostasis and by synthesizing vitamins and amino acids [4,5]. A disturbed composition of the gut microbiome has been associated with the pathogenesis of diseases such as inflammatory bowel disease, chronic inflammation, dyslipidemia, diabetes mellitus (DM), atopic disorders, cardiovascular disease (CVD), neoplasms and obesity [6,7,8,9,10].

Evidence of a link between the gut microbiota and renal function is rare; however, very recently, the role of the gut microbiota in renal physiology and pathophysiology has been investigated [11,12,13]. The normal gut microbiota plays an important role in the protection of the kidney, while gut dysbiosis might lead to the development of chronic kidney disease (CKD) [14]. The gut microbiome composition is considerably changed in CKD [15], promoting bacterial families that possess urease, uricase, indole and p-cresol-forming enzymes [16,17,18,19]. A cascade of abnormalities occurs owing to gut dysbiosis, such as accumulation of uremic toxins, systemic inflammation and infection, which play a role in the development of CKD and the deterioration of the renal function [20,21,22,23,24,25].

It has been reported that gut dysbiosis in CKD includes an increase in the species of *Entrobacteraceae* and *Pseudomonadaceae* genera of the phyla Proteobacteria, Bacteroidaceae and Clostridiaceae and a decrease in species of Lactobacillaceae, Prevotellaceae and Bifidobacteriaceae [15]. In end-stage kidney disease (ESKD), a total of 190 bacterial operational taxonomic units (OTU)— phyla Firmicutes, Actinobacteria and Proteobacteria—are increased [15]. Moreover, a growing number of studies support that probiotics such as *Lactobacillus* and *Bifidobacterium* in patients with CKD may lead to a decrease in uric acid, blood urea nitrogen (BUN) [26,27], serum creatinine [26] and estimated glomerular filtration rate (eGFR) [28].

While epidemiological studies are limited because they are not able to rule out the chance of residual bias, confounding factors and reverse causation, Mendelian Randomization (MR) analysis is able to compensate for these limitations, when applied to both phenotypes and genotypes [29]. A recent study reported that the levels of *Desulfovibrionaceae*, *Bacteroidaceae*, *Alcaligenaceae*, *Pseudomonadaceae*, and *Pasteurellaceae* (producing urease) were much lower in ESKD patients than in normal individuals, while *Bacteroidaceae* relative abundance was higher in these patients [30]. Reports based on a Chinese population with CKD revealed that the amount of *Bacteroides* was positively correlated with the level of creatinine and dominant in ESKD patients [30], whereas *Faecalibacterium* was positively associated with eGFR and dominant in controls [30]. Moreover, *Bifidobacterium* was negatively associated with BUN and creatinine [30]. These limited and mainly small observational studies, however, are difficult to interpret because they are influenced by confounding factors, such as diet, which may affect the gut microbiota and health, and by changes in the gut microbiota in response to illness. A recent animal study reported that increased urea concentration may be a leading contributor to dysbiosis changes in CKD and that both serum creatinine and BUN increased around 2.5-fold in animals with CKD [31]. Furthermore, the CKD group exhibited a considerably lower relative abundance of *Anaerostipes* spp., as well as an increased relative abundance of *Akkermansia* spp. and *Lactobacillus* spp. [31].

Taking the abovementioned factors into account, we conducted an MR analysis. This method is able to circumvent the limitations of observational studies [29] by using genetic variants that are associated with a type of exposure (here, microbiota genera) as instruments to test for associations with an outcome (here, risk of CKD and renal dysfunction); further genetic endowment is randomly allocated at conception, analogous to the randomization in randomized controlled trial (RCT) [29]. To the best of our knowledge, this is the first MR study on the role of the gut microbiota in renal function. We performed MR based on genome-wide association studies (GWAS) predicting eight genera applied to large extensively genotyped studies of risk of CKD and renal function to identify genera associated with these outcomes.

## 2. Methods

### 2.1. Study Design

A two-sample MR study design was used. Summary statistics were obtained from the largest GWAS on microbiota and outcomes of interest. We applied methods to estimate the unbiased effect of eight microbiome genera on the risk of CKD and renal function parameters (eGFR) separately in non-DM and DM participants.

### 2.2. Genetic Predictors of Exposures

From all GWAS, published on different microbiota genera, we used data for genera which had ≥2 single-nucleotide polymorphisms (SNPs): *Acidaminococcus* [32], *Anaerostipes* [33,34], *Bacteroides* [34], *Bifidobacterium* [33,34], *Desulfovibrio* [32], *Faecalibacterium* [32,34], *Lactobacillus* [32], *Megamonas* [32]. More information can be found elsewhere [32,33,34].

### 2.3. Genetic Predictors of Outcomes

Genetic associations with renal function were obtained from the largest available extensively genotyped study based on a meta-analysis (133,413 individuals with replication in up to 42,166 individuals) [35]. eGFR was estimated using the 4-variable Modification of Diet in Renal Disease Study Equation [35]. CKD was defined as eGFR < 60 mL/min/1.73 m^2^. DM was defined as fasting glucose ≥126 mg/dl, with pharmacologic treatment for DM or self-reported treatment [35]. In all studies, DM and kidney function were assessed at the same time point [35]. For genome-wide association analysis, a centralized analysis plan was followed; each study regressed sex- and age-adjusted residuals of the logarithm of eGFR on SNP dosage levels [35]. Logistic regression of CKD status was performed on SNP dosage levels adjusting for sex and age. For all traits, adjustment for appropriate study-specific features, including study site and genetic principal components, was included in the regression, and family-based studies appropriately accounted for relatedness [35]. An SNP, highly correlated (*R2* > 0.99) with the original SNP, was used as proxy when the original SNP was not available for outcomes.

### 2.4. MR Analysis

We combined the effect of 3 instruments using the inverse-variance weighted (IVW) method as implemented in the TwoSampleMR package running under R. We assessed heterogeneity using the Q value for the IVW method. To address the potential effect of pleiotropic variants on the final effect estimate, we conducted sensitivity analysis including weighted median (WM) and MR-Egger. Sensitivity analysis was conducted using the leave-one-out method. WM estimate, as the weighted median of the SNP-specific estimates, provides correct estimates as long as SNPs accounting for ≥50% of the weight are valid instruments. WM MR allows some variants to be invalid instruments provided at least half are valid instruments. It uses IVW and bootstrapping to estimate confidence intervals (CIs) [36]. MR-Egger has the ability to make estimates by the assumption that all SNPs are invalid instruments as long as the assumption of instrument strength independent of direct effect (InSIDE) is satisfied [36]. MR-Egger allows free estimation of the intercept, although further assumptions, such as the independence between instrument strength and direct effects, cannot be easily verified. Average directional pleiotropy across genetic variants was assessed from the *p*-value of the intercept term from MR-Egger [36]. Causal estimates in MR-Egger are less precise than those obtained by using IVW MR [37]. Analysis using MR-Egger has a lower false positive rate but a higher false negative rate than IVW [38].

Further, to assess heterogeneity between individual genetic variant estimates, we used the Q’ heterogeneity statistic [39] and the MR pleiotropy residual sum and outlier (MR-PRESSO) test [39]. The Q’ statistic uses modified 2nd-order weights that are a derivation of a Taylor series expansion and take into account uncertainty in both numerator and denominator of the instrumental variable ratio (this eases the no-measurement-error [NOME] assumption) [39]. The MR-PRESSO framework relies on the regression of variant–outcome associations on variant–exposure associations and implements a global heterogeneity test by comparing the observed distance (residual sums of squares) of all variants to the regression line with the distance expected under the hypothesis of no pleiotropy [40,41]. In case of evidence of horizontal pleiotropy, the test compares individual variants expected and observed distributions to identify outlier variants. We also applied the MR-Robust Adjusted Profile Score (RAPS), which allows correcting for pleiotropy using robust adjusted profile scores. We considered as results causal estimates that agreed in direction and magnitude across MR methods, passed nominal significance in IVW MR, and did not show evidence of bias from horizontal pleiotropy using heterogeneity tests. We used R (version 3.4.2 R Core Team, 2017).

### 2.5. Ethics

This investigation used published or publicly available summary data with no identification of the participants. No original data were collected for this manuscript. Ethical approval for each of the studies included in the investigation can be found in the original publications (including informed consent from each subject). The study conforms to the ethical guidelines of the 1975 Declaration of Helsinki.

## 3. Results

Instruments have F statistics higher than threshold, so that significant bias due to the use of weak instruments is unlikely [42]. Based on the data of two SNPs from different GWAS, *Anaerostipes* was associated with a higher eGFR in the overall population (IVW: β = 0.003, *p* = 0.021, Table 1) and non-DM subgroup (IVW: β = 0.003, *p* = 0.033, Table 1), while it appeared to have no significant effect on the risk of CKD (IVW: β = 0.009, *p* = 0.672, Table 1) and on eGFR in DM subjects (IVW: β = 0.005, *p* = 0.312, Table 1). With regard to *Desulfovibrio*, on the basis of two uncorrelated SNPs from the same GWAS, the analysis of the total population with higher abundance of the *Desulfovibrio* showed significant negative association with eGFR (IVW: β = −0.001, *p* = 0.035, Table 1); the same was found in non-DM subjects (IVW: β = −0.001, *p* = 0.007, Table 1), while we found no significant association between Desulfovibrio and risk of CKD (IVW =, β = 0.016, *p* = 0.191, Table 1) and eGFR value in DM subjects (IVW =, β = 0.0042, *p* = 0.157, Table 1). The results of heterogeneity are shown in Table 1. We found that none of the estimated associations was subjected to a significant level of the heterogeneity (for all IVW, *p* > 0.110, Table 1). The results of MR-RAPS were identical with the IVW estimates, which indicted no chance of pleiotropy (Table 1).

We also evaluated the impact of *Acidaminococcus*, *Bacteroides*, *Bifidobacterium*, *Faecalibacterium*, *Lactobacillus* and *Megamonas* on eGFR and risk of the CKD and showed no significant effect on renal function in the total population, DM and non-DM subgroups (for all IVW: *p* > 0.105); they had also no significant effect on the risk of CKD (IVW: *p* > 0.201 for all).

The results of heterogeneity indicated no chance of heterogeneity (*p* > 0.322 for all comparisons). The analysis of MR-PRESSO did not highlight any outlier for all estimates. The pleiotropy test (for those genera with more than two SNPs), with very negligible intercept and insignificant *p*-value, also indicated no chance of pleiotropy for all our estimations (*p* > 0.253 for all). The results of MR-RAPS were identical with the IVW estimates, which again indicated no chance of pleiotropy. The leave-one-out method demonstrated that the links were not driven by a single SNP.

## 4. Discussion

We conducted an MR analysis to evaluate the association of different microbiota genera with renal function and risk of CKD. Our results highlighted that subjects with genetically higher level of the *Anaerostipes* genus had a higher eGFR and better kidney function, while those with higher abundance of the *Desulfovibrio* genus has a lower eGFR. There was no evidence that pleiotropy, heterogeneity or outliers had biased these results.

Alterations of the intestinal barrier and change in the composition of the gut microbiota are leading factors contributing to intestinal dysbiosis [43,44]. Several alterations of the intestinal microbiota have been reported in CKD/ESKD, such as increased number of aerobic microorganisms as well as aerobic bacterial overgrowth (Proteobacteria and Actinobacteria) and the overgrowth of anaerobic bacteria, including Firmicutes [15,45].

Some important pathophysiological hypotheses have evolved with regard to the relationship between microbiota and renal dysfunction [23]. As an example, production and accumulation of toxic end products derived from bacterial fermentation and translocation of endotoxins and bacteria from the gut lumen into the blood stream can trigger chronic inflammation and worsen uremia toxicity [23]. Uric acid is excreted in the urine via a complex interplay of glomerular filtration and tubular reabsorption and secretion [46,47]. Moreover, as diet has an important role in the composition of the gut microbiota, a strict restriction of fruits and vegetables consumption (that is routinely prescribed for subjects with renal dysfunction) due to their potassium and oxalate content could deteriorate the structure or metabolism of the gut microbiota [15]. Further, long-term consumption of phosphate-binding agents by advanced CKD patients can change the luminal milieu of the gut; in turn, the abundance of the resident gut microbiota can be affected [15]. The use of antibiotics by CKD patients is a major factor modifying the composition and function of the gut microbiota [15].

Recent studies have reported the detrimental effects of uremia on the gut barrier structure and function [48,49,50]. Urea accumulation is one of the main factors in CKD, leading to increase urea influx into the intestinal lumen. Microbial urease hydrolyses urea to ammonia—ammonium hydroxide— which enhances the influx of inflammatory leukocytes leading to the production of local cytokines that induce retraction and endocytosis of transcellular tight junction proteins (claudins and occludin) [51,52]. Vaziri et al. found differences in the intestinal microbiota between uremic and non-uremic patients and reported that uremia contributed to this by increasing intestinal disease-causing bacteria [15]. Studies show that the gut microbiome community is considerably changed in CKD [15], because there is a dominance of bacterial families that possess urease, uricase, indole and p-cresol-forming enzymes [16]. Of note, ESKD patients show decreased numbers of bacteria producing short-chain fatty acid (SCFAs). It has been speculated that SCFA have an anti-inflammatory role [16]. On the other hand, gut-derived uremic toxins such as indoxyl sulfate and p-cresyl sulfate induce pro-inflammatory responses and promote leukocyte stimulation [53,54], and these metabolites are associated with increased levels of inflammatory markers (e.g., interleukin-6 and glutathione peroxidase) in CKD patients [55,56]. It is known that inflammation is one of the main factors initiating renal dysfunction. Animal studies showed that there was a significant difference between nephrectomised rats and healthy ones, and it was found that 175 operational taxonomic units differed between the two groups; changes included a lower abundance of Bacteroidetes and Firmicutes, especially Lactobacillaceae and Prevotellaceae [15].

*Anaerostipes* are Gram-variable, obligate anaerobes producing acetate and butyrate [57]. Increased abundance of *Anaerostipes* may result in the increased production of SCFAs as well as in the decrease of branched-chain fatty acids in the gut and subsequently in the blood [58]. Bacteria producing SCFAs, especially butyrate, are decreased in ESKD patients [30]. Bacteroidetes (40%), Firmicutes (40%) and Proteobacteria (10%) are the predominant phyla in healthy individuals compared with CKD patients [30,59]. Butyrate produced from microbial fermentation plays an important role in the adjustment of the body reactions to inflammation [60]. Bacteria producing butyrate were negatively associated with inflammatory factors such as C-reactive protein (CRP) and renal function markers [30]. It has also been reported that SCFAs might decrease the chance of acute renal dysfunction by reducing inflammation [61]. There is no human study on the link between *Anearostipes* and eGFR; only one animal study is available [31]. C57BI/6J mice (with surgically induced CKD) underwent either a sham operation (Sham) or unilateral nephrectomy with simultaneous ipsilateral upper pole cautery resection (NPX) at eight weeks of age (*n* = 10 mice per group). Both serum creatinine and BUN increased around 2.5-fold in the NPX group compared with the Sham group [31]. The diversity of the microbial communities between these groups showed that NPX mice had lower abundance of *Anearostipes* spp. [31]. NPX mice with low abundance of *Anaerostipes* showed negative effects on kidneys parameters (BUN and creatinine) [31]. Furthermore, both urea supplementation and the experimental CKD model resulted in decreased relative abundance of *Anearostipes* [31]. The results of this study are somewhat consistent with those of our study, which showed that *Anearostipes* led to increased eGFR and improvement in renal function.

The *Desulfovibrio* genus includes sulfate-metabolizing bacteria that reduce the sulfites and sulfates obtained from the diet and the sulfated mucopolysaccharides found in mucin, leading to the generation of hydrogen sulfide, a cytotoxic compound [62]. A recent study involving 52 ESKD patients (mean age 51.6 ± 18 years) and 60 healthy individuals (mean age 52.5 ± 14 years) that evaluated the alteration of the gut microbiota in a Chinese population revealed that *Desulfovibrio* spp. produced urease, whose relative abundance was much lower in ESKD patients than in the control group [30]. It was further reported that *Desulfovibrio* spp. clearly flourish in an inflammatory environment [62], and harmful effects of inflammatory factors on the progression of renal dysfunction have been discussed [63,64,65].

Several mechanisms have been proposed for the detrimental effect of *Desulfovibrio* spp. on kidney function. The main theory centres on the fact that SCFAs such as butyrate are metabolized by *Desulfovibrio*. Then, *Desulfovibrio* uses sulfites and sulfates as terminal metabolic electron acceptors, leading to the generation of hydrogen sulfide, which has a wide range of cytotoxic effects. Among these, hydrogen sulfide is a potent inhibitor of the oxidation of SCFAs in cells, thereby completing a vicious circle of mutually exclusive metabolic interactions between host and this bacterium [62].

With regard to diabetic populations, we did not find an association of *Desulfovibrio* and *Anearostipes* with eGFR and CKD. It should be noted that different mechanisms and factors in diabetic and non-diabetic individuals may explain why we did not find any association in the diabetic subgroup [7]. Oxidative stress and inflammatory markers are suggested to be associated with macrovascular and microvascular complications of diabetes [8]. CKD as one of the long-term complications of diabetes is related to oxidative stress [7]. It could be hypothesized that high levels of oxidative stress and inflammatory markers in diabetic patients might be confounding factors affecting any association. Also, there are other factors which we did not consider in our study. We can only make assumptions in explaining our findings; the exact mechanisms are unknown, and more studies considering more confounding factors are required.

Our analysis has strengths and limitations. MR is a powerful tool for the detection of causal impacts, which is an advantage with respect to observational studies. Moreover, we benefited from the largest GWAS on exposure and outcomes. However, the MR analysis has limited statistical power. Therefore, a lack of findings in our analysis might be due to small causal effects that were not detectable in our study. Our study used genetic instruments and estimation of genetic associations using GWAS that were mainly based on a sample of European ancestry. It is known that the microbiota genera vary by ethnicity and other factors. Therefore, our results cannot be extrapolated to other ethnicities. We could not test for subgroup analysis (e.g., by age and sex) and diet–microbiome interactions, but causal effects should be generally consistent. Second, the 16S ribosomal RNA gene sequencing used by most microbiota GWAS usually only permits resolution at the genus level rather than at a more specific level, so we cannot rule out the possibility that some specific species or strains are associated with renal function. Moreover, for future studies, it is important to consider the ratio between two taxa or dysbiosis of the gut microbiota. Additionally, the gut microbiota may also be influenced by other factors, such as age and season. However, the gut microbiota is thought to be stable especially after early childhood, and the main force determining its composition consists of long-term dietary habits. As such, our findings may be more relevant to the effects of the gut microbiota in adolescence or adulthood. We would like to call for more GWAS with greater sample size to include other bacterial genera (we understand that the validity of SNPs might be low because of the nature of the original GWASs). Our study is also limited by the current understanding of the gut microbiota. A hypothesis-driven study testing epidemiologically established associations would have been preferable. However, this was precluded by the lack of knowledge as to the function of each microbiome constituent and the lack of large epidemiological studies. However, for both genera which had an effect on renal function, there were experimental studies enabling us to discuss possible mechanisms.

In conclusion, our study highlighted the association of *Desulfovibrio* and *Anearostipes* spp. on renal function markers. Further studies using multiple robust instruments are needed to confirm these results.

## Figures and Tables

**Table 1:** Results of mendelian randomization analysis for all exposures.

Exposures		MR				Heterogeneity		
		Method	Beta	SE	*p*	Method	Q	*p*
*Anaerostipes*	CKD	IVW	0.009	0.022	0.672	IVW	0.237	0.626
		RAPS	0.009	0.022	0.681			
	eGFR	IVW	0.003	0.002	0.021	IVW	2.548	0.110
		RAPS	0.003	0.001	0.032			
	eGFR (Non-DM)	IVW	0.003	0.001	0.033	IVW	1.895	0.168
		RAPS	0.003	0.001	0.015			
	eGFR (DM)	IVW	0.005	0.005	0.312	IVW	0.256	0.612
		RAPS	0.005	0.005	0.329			
*Desulfovibrio*	CKD	IVW	0.016	0.012	0.191	IVW	0.007	0.931
		RAPS	0.016	0.013	0.208			
	eGFR	IVW	−0.001	0.0007	0.035	IVW	0.168	0.681
		RAPS	−0.001	0.0007	0.047			
	eGFR (Non-DM)	IVW	−0.001	0.0007	0.007	IVW	0.077	0.780
		RAPS	−0.002	0.0008	0.013			
	eGFR (DM)	IVW	0.0042	0.0029	0.157	IVW	0.792	0.373
		RAPS	0.0042	0.0031	0.172			

Inverse-variance weighted, IVW, RAPS, Robust Adjusted Profile Score, SE, standard error, beta, beta-coefficients, MR, Mendelian randomization, Q, Q value for heterogeneity statistic, CKD, chronic kidney disease, eGFR, estimated glomerular filtration rate, DM, diabetes mellitus.
